# Imaging Shock Waves in Diamond with Both High Temporal and Spatial Resolution at an XFEL

**DOI:** 10.1038/srep11089

**Published:** 2015-06-18

**Authors:** Andreas Schropp, Robert Hoppe, Vivienne Meier, Jens Patommel, Frank Seiboth, Yuan Ping, Damien G. Hicks, Martha A. Beckwith, Gilbert W. Collins, Andrew Higginbotham, Justin S. Wark, Hae Ja Lee, Bob Nagler, Eric C. Galtier, Brice Arnold, Ulf Zastrau, Jerome B. Hastings, Christian G. Schroer

**Affiliations:** 1 Deutsches Elektronen-Synchrotron DESY, Notkestr. 85, D-22607 Hamburg, Germany; 2 Institute of Structural Physics, Technische Universität Dresden, D-01062 Dresden, Germany; 3 Lawrence Livermore National Laboratory, 7000 East Avenue, Livermore, CA 94550, USA; 4 Centre for Micro-Photonics, Swinburne University of Technology, Hawthorn, VIC 3122, Australia; 5 Department of Physics, Clarendon Laboratory, University of Oxford, Parks Road, Oxford OX1 3PU, United Kingdom; 6 Linac Coherent Light Source, SLAC National Accelerator Laboratory, 2575 Sand Hill Road, Menlo Park, CA 94025, USA

## Abstract

The advent of hard x-ray free-electron lasers (XFELs) has opened up a variety of scientific opportunities in areas as diverse as atomic physics, plasma physics, nonlinear optics in the x-ray range, and protein crystallography. In this article, we access a new field of science by measuring quantitatively the local bulk properties and dynamics of matter under extreme conditions, in this case by using the short XFEL pulse to image an elastic compression wave in diamond. The elastic wave was initiated by an intense optical laser pulse and was imaged at different delay times after the optical pump pulse using magnified x-ray phase-contrast imaging. The temporal evolution of the shock wave can be monitored, yielding detailed information on shock dynamics, such as the shock velocity, the shock front width, and the local compression of the material. The method provides a quantitative perspective on the state of matter in extreme conditions.

Whilst many of the advances afforded by x-ray free-electron lasers (XFELs)^1^,^2^,^3^ are completely new^4^,^5^,^6^, several key results have been obtained by exploiting established static techniques and transporting them to the femtosecond time-domain made possible by the short duration of the XFEL pulses^7^,^8^. Hard x-ray phase-contrast imaging (PCI), an established technique at modern synchrotron radiation sources, enables one to image optically opaque samples with high spatial resolution^9^,^10^,^11^. Its sensitivity to the phase shift introduced by an object enhances the visibility of structures otherwise invisible in x-ray radiography based on absorption^12^,^13^. When combined with the femtosecond duration of an XFEL pulse, such a technique allows imaging of matter changing rapidly in both space and time. We make use of the very short but intense XFEL pulses to image the propagation of an elastic compression wave in diamond with both high temporal (~50 fs pulse duration) and spatial resolution (~500 nm). As shock waves in matter typically travel at speeds in the range of several kilometers per second, x-ray pulses in the picosecond regime are required to freeze the motion of the shock wave. We developed a new x-ray microscope based on beryllium compound refractive x-ray lenses (Be CRLs)^14^, which is especially optimized for the XFEL environment and can withstand the full XFEL beam^15^. The x-ray microscope is part of an infrared-laser-pump-x-ray-probe setup. It generates shock waves in matter using a short optical laser pulse and probes the sample state using magnified phase-contrast imaging with a single XFEL pulse. From these images, we can quantitatively reconstruct the density profiles and thus infer the state immediately behind the shock front.

The extreme physical properties of diamond make it a material of enduring scientific and technological interest. Its unique combination of ultra-high stiffness, hardness, optical transparency, and thermal conductivity have made it a focal point of high-pressure science for decades. Several recent publications have examined the behaviour of diamond under dynamic compression from 0.1 TPa to 5 TPa^16^,^17^,^18^,^19^,^20^. All these studies used optical interferometry to infer conditions in the compression wave. Here for the first time we directly image the shock wave using x rays, opening up a valuable new perspective on the dynamic behaviour of diamond under high pressure.

The experiment was carried out at the Matter in Extreme Conditions (MEC) endstation of the Linac Coherent Light Source (LCLS) XFEL. The instrument is located in the far-experimental hall at a distance of about 464 m from the undulator x-ray source (cf. Fig. 1). The x-ray microscope is based on a magnified inline imaging scheme that requires the creation of a secondary source using x-ray optics^12^,^21^. At a photon energy of 8.2 keV a stack of 20 Be CRLs focuses the LCLS beam to a spot with a size of 125 nm full width at half maximum (FWHM) at a focal length of 250 mm behind the optics^22^.

The diamond samples (cf. Sample preparation in the Methods) were positioned at a distance of *z*_*s*_ = 115 mm behind the focus. The magnified near-field images of the samples were projected onto a detector located at *z*_*d*_ = 4214 mm behind the sample (cf. Fig. 1), yielding a magnification of *M* = 37.6^15^. The method requires a high degree of spatial but only a moderate degree of longitudinal coherence^23^. These requirements are well matched by the beam characteristics of an XFEL based on self-amplified spontaneous emission (SASE), such as those at the LCLS^24^,^25^. The shock wave was induced in the diamond samples by shining an 800 nm-infrared laser with a pulse duration of 150 ps (FWHM) onto the sample from the side (cf. Optical laser in the Methods).

Figure 2 shows a series of phase-contrast images measured at different time delays ranging from 1.2 ns in Fig. 2(a) to 3.0 ns in Fig. 2(d). These images clearly show the decay of the elastic compression wave during the 3 ns time interval producing less contrast towards the longer time delays. Since the target area is destroyed by the high-intensity optical laser pulse, each of these images was taken at a fresh spot on the sample or even different diamond samples.

To obtain quantitative measurements of the density in the shock front and its velocity we model the image formation process that leads to the images in Fig. 2(a–d). The transmission of the x rays through the thin object is described by a complex transmission function *O*(**r**) that relates the transmitted wave field *ψ*(**r**) to the wave field *P*(**r**) incident on the sample by *ψ*(**r**) = *O*(**r**) ⋅ *P*(**r**). The transmitted wave field then propagates to the detector. In the paraxial approximation the propagation is modeled by the Fresnel-Kirchhoff integral^26^. By recording the intensity in the detector plane, the phases of the x-ray wave field are lost. With the knowledge of the incident wave field obtained by an antecedent beam-characterization experiment (ptychography)^22^ the transmission function *O*(**r**) can be reconstructed by phase-retrieval techniques (cf. Data analysis in the Methods). In this way image distortions related to phase modulations in the incident wave front can be corrected for. Such distortions, which are due to aberrations of the lens or other defects, are visible for example in Fig. 2(b) (marked by arrows). The compression of the material by the shock wave introduces an additional phase shift in the x-ray wave field behind the sample. Figure 2(e–h) depicts the phase maps [phase of the transmission function *O*(**r**)] in the sample plane obtained by iterative phase retrieval from Fig. 2(a–d), respectively.

Figure 3 shows line profiles through the shock front extracted from Fig. 2(e–h) in an area highlighted by a dashed rectangle in Fig. 2(f). These profiles monitor the compression of the material in the propagating shock wave at the different time delays. The signal was averaged over a length of 10 μm perpendicular to the propagation direction in order to reduce the influence of noise. By measuring the distance between the sample edge and the shock front, a shock velocity *v* = (19.9 ± 1.7) km/s was determined (cf. Magnified phase-contrast imaging in the Methods).

The phase profiles shown in Fig. 3 are well represented by a double-sided error function as indicated by dashed lines. Since the reconstructed phase change corresponds to an integrated value accumulated along the path of the x rays through the sample, it is in general not proportional to the local compression of the material. In order to retrieve the compression quantitatively, further modeling of the shock wave inside the material is required. To this end, we assume a spherically curved wave front whose radius of curvature can be obtained from Fig. 2. Using tomographic reconstruction under the assumption of spherical symmetry, the local phase change Δ*φ* per voxel is reconstructed (cf. Data analysis in the Methods). In Fig. 4 line profiles monitoring the local change in density are summarized. They were obtained from the tomographically reconstructed phase profiles Δ*φ* by using Δ*ρ* = −Δ*φ*/[*kd*(*δ*/*ρ*)], where Δ*ρ* is the change in density, *k* = 4.16 × 10^7^ mm^−1^ the wave number, *d* = 59.8 nm the thickness of a cubic voxel, and *δ*/*ρ* = 3.1 × 10^−6^ (g/cm^3^)^−1^ the refractive index decrement *δ* normalized to the density *ρ* of the uncompressed material at the photon energy of *E* = 8.2 keV.

As the compression wave propagates into the target (cf. Fig. 4), it decreases in amplitude and increases in width. This is due to the finite duration of the originally applied pressure pulse, which results in a rarefaction wave that releases the stress behind the shock front. In Fig. 5(a) the decay of the maximum compression *C*_max_ as function of time *t* is shown. Specific compression values were determined using *C*_max_(*t*) = Δ*ρ*_max_(*t*)/*ρ*, with *ρ* = 3.52 g/cm^3^ the bulk density of diamond. At the same time the shock width increases from about 1.5 μm to 3.5 μm in the given time window [cf. Fig. 5(b)]. Error bars in Fig. 5(b) indicate an 8.7% inaccuracy in shock width, which is related to the error in length scale in the phase-contrast images (cf. Magnified phase-contrast imaging in the Methods). The high sensitivity of PCI allows one to measure density variations as small as 1% in this case, and the early time steepening of the wave front is resolved for the first time with a sub-micron resolution. Numerical calculations confirm that for the applied pressure pulse the compression steepens up within the first nanosecond owing to the non-linear compressibility, such that the front itself has a width of order the lattice spacing. Although this sharp front can not be resolved with this setup, it can be used to determine the resolution power of the microscope. From the inferred compression, we conclude that this is a purely elastic wave, as the pressures are well below the Hugoniot elastic limit (HEL) of diamond^18^.

In this first approach to quantitatively analyze the phase-contrast images we determined a wave curvature of *R* = 440 μm at the time delay of Δ*t* = 1.2 ns, which scales linearly up to *R* = 476 μm at Δ*t* = 3.0 ns. However, as can be seen in Fig. 2(e–h) the precise shape of the elastic wave varies considerably between different pulses and, therefore, we make a conservative error estimate for the radius of curvature of approximately Δ*R*/*R* ≈ 30%. Numerical simulations showed that this systematic error propagates into the determination of the material induced phase shift using tomography by Δ*φ*/*φ* ≈ 0.5 ⋅ Δ*R*/*R* = 15%. Since density change Δ*ρ* and compression *C* are linearly related to this phase change, the same relative error holds for the deduced values, i. e., Δ*ρ*/*ρ* = Δ*C*/*C* ≈ 15% [cf. error bars in Fig. 5(a)].

With this example, we demonstrated that fast dynamic processes inside matter can be visualized *in situ* with both high temporal and high spatial resolution by magnified x-ray phase-contrast imaging at an XFEL. Smallest visible features had a size of about 500 nm, mainly limited by the bandwidth of the XFEL beam. A seeded XFEL beam combined with an optimized detector setup will improve the resolution to better than 100 nm. Note that the temporal resolution, set by the x-ray pulse length, is shorter than even the fastest phonon period in the system, totally eliminating temporal blurring. This combination of spatial and temporal resolution could thus find a range of applications in shock physics, as this scale length is, for example, smaller than typical grain sizes in most polycrystalline samples, affording the possibility of observing the effect of grain boundaries and orientations on shock propagation. Furthermore, although the elastic compression wave here was atomic in front width, the direct observation of the evolution of the width of the front in a shock for a material subjected to pressures above the HEL would provide significant insight into the time scales for plastic flow under high strain-rate conditions well in excess of 10^9^ s^−1^.

## Methods

### Diamond samples

The diamond samples are optical grade polycrystalline strips with dimensions of Δ*x* × Δ*y* × Δ*z* = 30 mm × 145 μm × 272 μm. They are produced by chemical vapor deposition (CVD) and contain micrometer-sized grains. The LCLS beam, propagating in 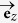-direction, hits the polished side of the sample having less than 7 nm peak-to-valley roughness, whereas the shock-driving laser pulse is incident perpendicular to the LCLS beam onto the unpolished side of the sample propagating in 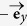-direction. The elastic wave therefore propagates in 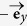-direction. The x-ray transmission image is recorded through the 272 μm-thick side of the sample.

### Optical laser

The shock wave was initiated by a Gaussian-shaped, 150 ps (FWHM) drive laser with a wavelength *λ* = 800 nm and energy *E* = 130 mJ per pulse. The optical beam was focused onto the diamond target to a spot with a size of 80 μm (FWHM), which corresponds to a maximum peak power of *P* ≈ 12 TW/cm^2^.

### Magnified phase-contrast imaging

The experiment was carried out in a magnified inline geometry. By using a set of 20 Be CRLs a secondary x-ray source was created at *z*_*s*_ = 115 mm in front of the sample^22^. The area of the sample illuminated by the divergent x-ray beam was imaged onto a high-resolution x-ray detector positioned at a distance of *z*_*d*_ = 4214 mm after the sample, resulting in a magnification of *M* = (*z*_*s*_ + *z*_*d*_)/*z*_*s*_ = 37.6. The scintillator-based high-resolution detector has an effective pixel size of *p*_*d*_ = 2.25 μm, leading to *p* = *p*_*d*_/*M* = 59.8 nm pixels in the phase-contrast images. This experimental arrangement with spherical-wave illumination is equivalent to a plane wave geometry with an effective propagation distance of *z*_eff_ = *z*_*d*_/*M* = 112 mm^12^,^13^,^27^. The error in magnification Δ*M* is related to measurement inaccuracies of the distance values *z*_*s*_ and *z*_*d*_. It is given by Δ*M*/*M* = |*z*_*d*_/(*z*_*s*_ + *z*_*d*_)| ⋅ (Δ*z*_*s*_/*z*_*s*_ + Δ*z*_*d*_/*z*_*d*_) ≈ Δ*z*_*s*_/*z*_*s*_ + Δ*z*_*d*_/*z*_*d*_, if *z*_*s*_ ≪ *z*_*d*_. In this case, it further reduces to Δ*M*/*M* ≈ Δ*z*_*s*_/*z*_*s*_, since the relative error in *z*_*d*_ was much smaller than the error in *z*_*s*_. The distance *z*_*s*_ could be calibrated with an accuracy of approximately Δ*z*_*s*_ = 10 mm, yielding a relative error in magnification and the corresponding length scale in the phase-contrast images of 8.7%. Since the shock velocity was determined by measuring the distance between the shock front and sample edge, this error propagates into the determination of the shock velocity, yielding finally *v* = (19.9 ± 1.7) km/s. Timing inaccuracies can be neglected, since the pulse-to-pulse jitter was only 300 fs and long-term drifts were smaller than 1 ps.

The spatial resolution of the method is mainly limited by the pixel size of the detector and the bandwidth of the incoming x-ray radiation. The slight polychromaticity reduces the contrast for high spatial frequencies. For a SASE bandwidth of Δ*λ*/*λ* = 2 ⋅ 10^−3^, we expect a reduction in phase-contrast transfer by 50% or more for a spatial frequency above *u*_max_ = 5440 mm^−1^, corresponding to a length scale of *d*_min_ ≈ 200 nm^28^. In addition, the chromaticity of the refractive lens leads to a slight blur (Δ*f* ≈ 1 mm) of the focus position along the optical axis^29^. Whereas this effect does not reduce the phase contrast for sample features located close to the optical axis, the smearing of the phase-contrast image becomes stronger towards larger angles and reaches a maximum of approximately 600 nm at the edge of the circular field of view.

### Data analysis

The reconstruction scheme is based on the Hybrid-Input-Output (HIO) algorithm^30^,^31^, making use of the additional knowledge of the complex-valued illumination function *P*(**r**) characterized by ptychography in an antecedent experiment^22^,^32^. The knowledge of *P*(**r**) is necessary to disentangle contrast introduced by the sample from wave-field structures already present in the incoming x-ray beam. Like computational spectacles, it corrects the spherical aberration of the Be CRLs. As described in the previous section, the conical imaging geometry is equivalent to a parallel-beam geometry with a propagation distance of *z*_eff_ = *z*_*d*_/*M* = 112 mm between object and detector plane.

**Figure 2:**
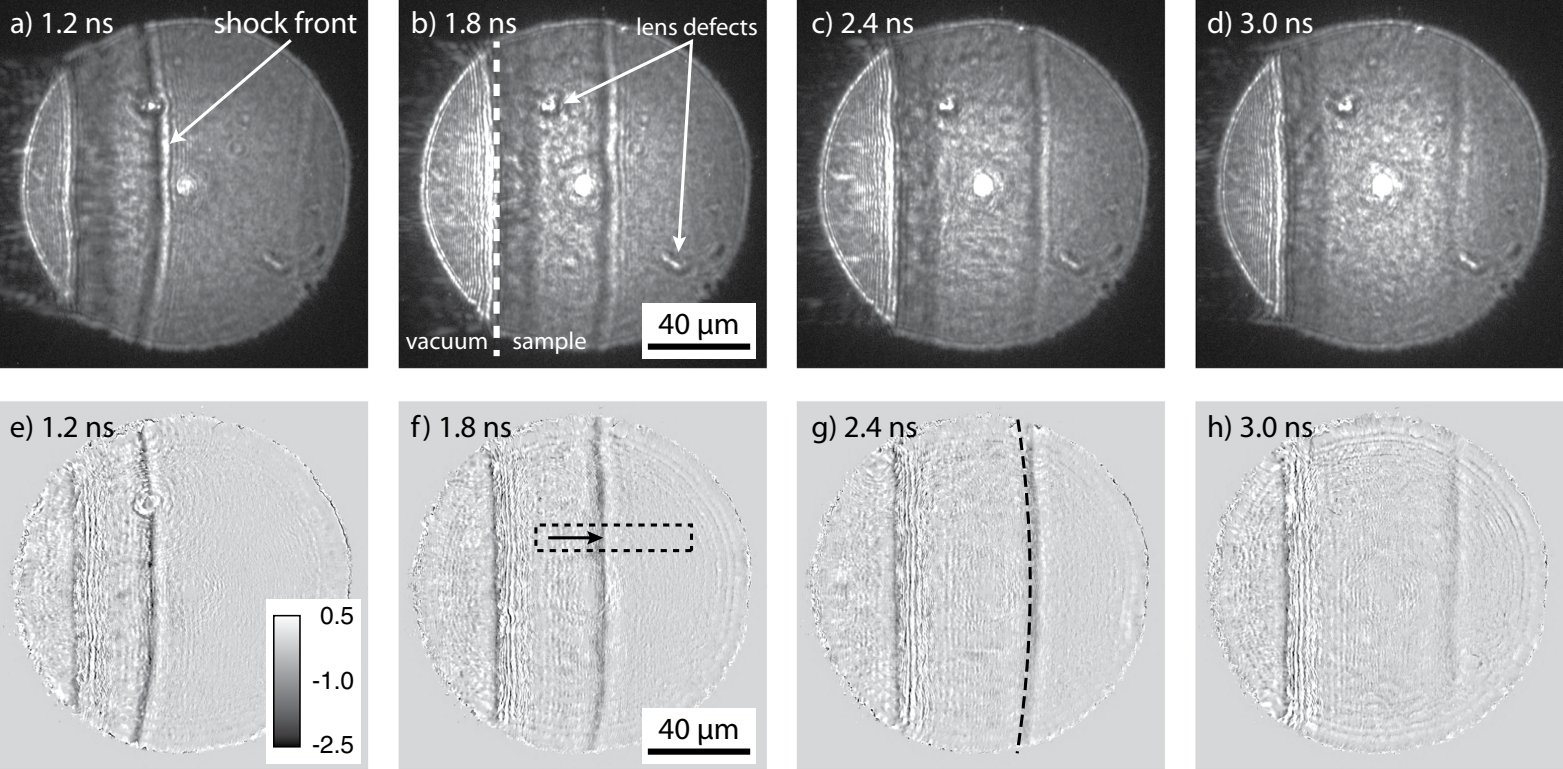
(**a**–**d**) Phase-contrast images measured with a high-resolution x-ray detector at a distance of 4214 mm behind the sample. Specific time delays are indicated in each image. (**e**–**h**) Corresponding phase maps obtained by iterative phase retrieval from the images above. In order to enhance the visibility of shock-related features, the phase map obtained from just the sample without shock wave was subtracted from the phase map with shock wave. Gray values indicate the phase shift in radians [cf. inset in Fig. e)]. In Fig. f) a rectangular box highlights the area used to quantitatively determine the compression of the material.

We reconstruct the object-transmission function *O*(**r**) iteratively: as a first guess *O*_1_(**r**) we use the reconstructed hologram. It is obtained by propagating the reconstructed probe function *P*(**r**) (obtained with ptychography) to the image plane, subsequent pointwise multiplication with the real-valued phase-contrast image *I*(**r**), propagating it back to the sample plane, and dividing the result by the complex probe function, i. e., 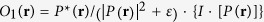, where 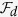 is the Fresnel propagator in paraxial approximation and *ε* a small regularizing constant. The central part of the phase-contrast image that contains a strong contribution from higher-harmonic radiation [bright spot at the center of Fig. 2(a–d)] was masked and set constant.

This step is followed by an iterative refinement of the object-transmission function based on a ptychographic algorithm^33^, but using just a single phase-contrast image. The complex transmission function behind the sample in a certain iteration *j* is given by


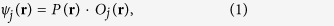


which is then propagated to the image plane


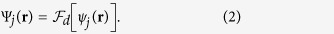


Here, the amplitude of the wave field is replaced by the measured phase-contrast imaging data *I*(**r**)


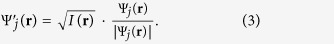


The back propagation to the sample plane yields an updated transmitted wave field


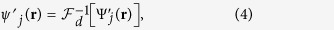


and the updated object function *O*_*j*+1_ is obtained by^33^


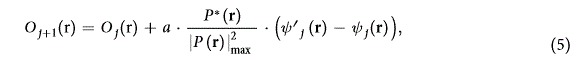


where the constant parameter *a* was set to 1. In a last step, the phase of the object function was constrained to the interval [−*π*,0] and, since the sample was composed of just a single material, the amplitude *A*(**r**) of the object function was moderately coupled to the phase shift *φ*(**r**)^34^. If *O*(**r**) = *A*(**r**)*e*^*iφ*(**r**)^, then


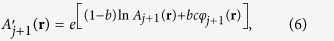


where *b* = 0.6 was set constant, and *c* = *β*/*δ* defines the material dependent coupling parameter between absorption and refraction. The parameters *δ* = 1.09 × 10^−5^ and *β* = 1.58 × 10^−8^ describe the refractive index decrement and the attenuation in the refractive index *n* = 1 − *δ* + *iβ* for diamond at *E* = 8.2 keV, respectively. Convergence was achieved after 100 iterations.

This procedure was carried out for phase-contrast images of the sample without and with the elastic shock wave at different time delays in the pump-probe experiment. The phase map without the elastic wave was then subtracted from the phase map with the shock wave. The resulting phase maps are shown in Fig. 2(e–h) and were the basis for further analysis.

**Figure 3:**
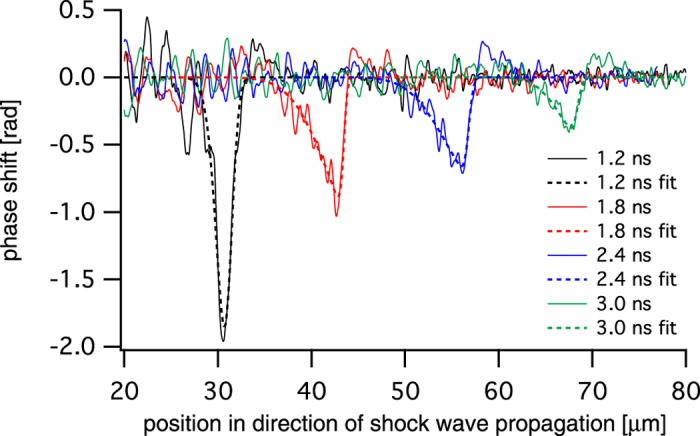
Phase profiles of the shock front at different time delays.

The extracted line profiles were fitted by a double sided error function of the form 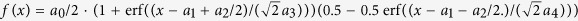, with fit parameters *a*_0_, *a*_1_, *a*_2_, *a*_3_, and *a*_4_ (cf. dashed lines in Fig. 3). The function well represents the extracted phase profiles and specific fit parameter values are summarized in Table 1.

In order to retrieve quantitative compression values the line profiles were further evaluated by standard tomographic techniques assuming a spherical distribution of compressed material. The curvature of the wave was determined directly from the image as indicated by a dashed line in Fig. 2(g). This assumes that the elastic wave is curved isotropically. In general, this assumption may be invalid, for example, when the density distribution is more complex. Here, however, the data do not indicate such a behavior. In the future, we want to investigate anisotropy effects in more complex materials.

**Figure 4:**
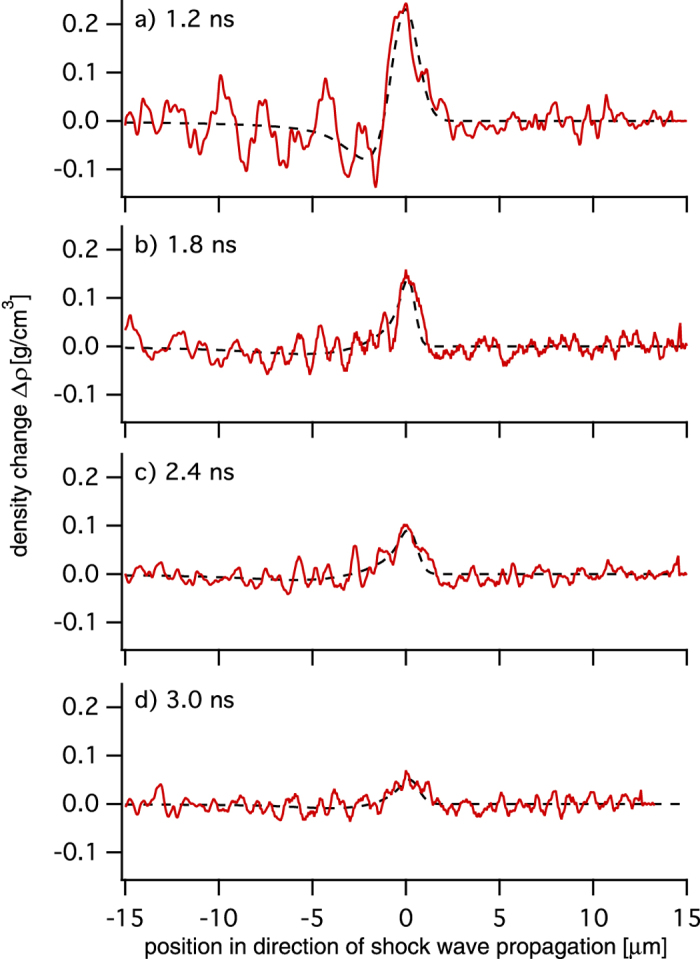
Density profiles of the shock front after the application of the tomographic reconstruction algorithm.

The phase profiles and their fits, which are shown in Fig. 3, served as input data for tomographic reconstruction that finally yields the phase shift per cubic voxel with a volume of (59.8)^3^ nm^3^. This phase shift can then be transferred into compression Δ*ρ*/*ρ* as explained in the main text. The result of the tomographic analysis is summarized in Fig. 4. The red solid lines refer to data profiles and dashed black lines to the corresponding fit data after application of the tomographic algorithm.

Supplemental movies illustrate the path of data analysis. The movie ‘diamond_pci.mov’ shows the raw data. In file ‘diamond_rec_sub.mov’ the reconstructed phase maps after iterative refinement are summarized. In the reconstructed images the phase maps without shock wave were subtracted from the actual shock measurements enhancing the contrast for shock features.

## Additional Information

**How to cite this article**: Schropp, A. *et al*. Imaging Shock Waves in Diamond with Both High Temporal and Spatial Resolution at an XFEL. *Sci. Rep*. **5**, 11089; doi: 10.1038/srep11089 (2015).

## Supplementary Material

Supplementary Movie S1

Supplementary Movie S2

## Figures and Tables

**Figure 1:**
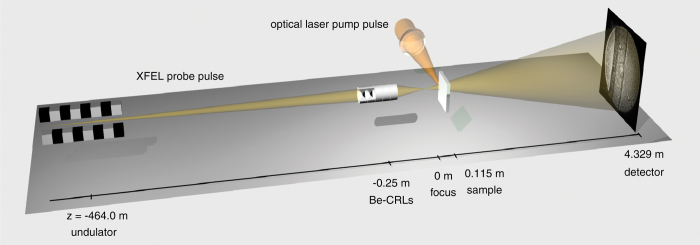
Schematic outline of the optical setup used for magnified x-ray phase-contrast imaging. Optical axis is not to scale.

**Figure 5:**
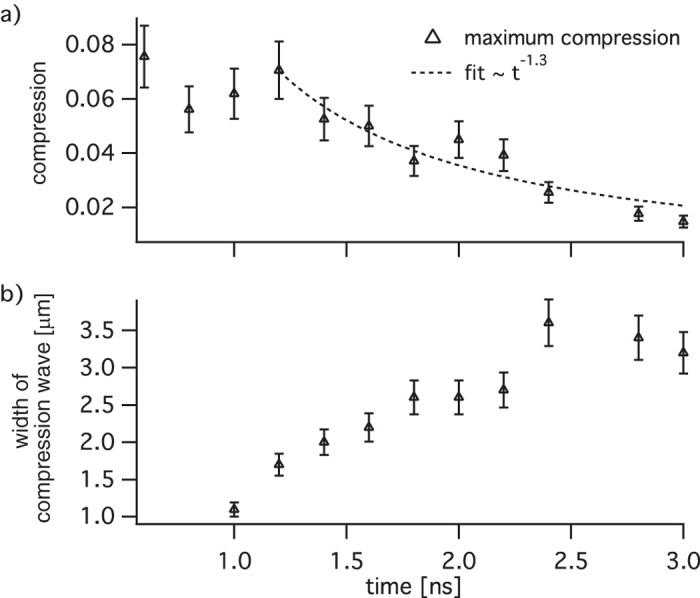
Decay of the elastic compression wave.

**Table 1:** Summary of fit parameters.

time delay	*a*_0 _[rad]	*a*_1_ [µm]	*a*_2_	*a*_3_	*a*_4 [µm]_
1.2 ns	−6.43	31.1	0.06	1.37	0.7
1.8 ns	−1.59	42.8	1.23	3.45	0.3
2.4 ns	−1.00	55.7	2.30	3.29	0.4
3.0 ns	−0.62	67.7	1.56	2.35	0.1
